# Characterization of extended-spectrum-*β*-lactamase producing *Klebsiellapneumoniae* phage KP1801 and evaluation of therapeutic efficacy in vitro and in vivo

**DOI:** 10.1038/s41598-020-68702-y

**Published:** 2020-07-16

**Authors:** Phitchayapak Wintachai, Ampapan Naknaen, Jirapath Thammaphet, Rattanaruji Pomwised, Narumon Phaonakrop, Sittiruk Roytrakul, Duncan R. Smith

**Affiliations:** 1 School of Science, Walailak University, Nakhon Si Thammarat, 80161 Thailand; 2 Department of Microbiology, Prince of Songkla University, Songkhla, 90112 Thailand; 3 Proteomics Research Laboratory, Thailand Science Park, Pathum Thani, 12120 Thailand; 4 Institute of Molecular Biosciences, Mahidol University, Bangkok, 73170 Thailand

**Keywords:** Drug discovery, Microbiology

## Abstract

Extended spectrum *β* lactamase-producing *Klebsiella* *pneumoniae* (ESBL-KP) is being reported with high morbidity and mortality rates and is considered as the highest priority for new antimicrobial strategies. To develop an alternative antimicrobial agent, phage KP1801 with broad lytic activity was isolated. The genome of phage KP1801 was double stranded DNA of 49,835 base pairs, with a GC content of 50.26%. There were 75 putative open reading frames. Phage KP1801 was classified as being in the order *Caudovirales*, belonging to the *Siphoviridae* family. About 323 proteins were detected by shotgun proteome analysis. The phage inhibited biofilm formation and reduced pre-formed biofilm in a dose dependent manner. Scanning electron microscopic studies demonstrated a membrane damage of bacterial cells treated with phage, resulting in cell death. Prophylactic and therapeutic efficacies of the phage were evaluated in *Galleria* *mellonella*. Administration of ESBL-KP infection with phage significantly improved the survival of *G.* *mellonella*. The number of intracellular bacteria in larvae showed a significant decrease compared with untreated control while the number of phage increased. These studies suggested that phage KP1801 has the potential for development as an alternative for antibiotics and biocontrol agents against ESBL-KP infection.

## Introduction

Extended spectrum *β* lactamase enzymes are commonly detected in almost all multidrug-resistant *Klebsiella* *pneumoniae*^1^, which is an opportunistic Gram-negative pathogen associated with severe infections such as wound infection, urinary tract infection, septicemia, pneumonia, intra-abdominal infection, and septic shock^2^. ESBL producing *K.* *pneumoniae* (ESBL-KP) has been reported as the most frequently isolated pathogen in hospital-acquired infections and is associated with significant morbidity and mortality in intensive-care units. ESBL-KP has therefore been attracting increasing attention because of its resistance to a broad range of beta-lactam antibiotics, the most commonly used class of antibacterial agents^3–7^.

The ability to form a biofilm, one of the main virulence factors, is considered an essential step in ESBL-KP infection, producing a favorable environment for exchange of antibiotic resistance genes^8^. Previous reports have shown that the susceptibility of bacteria to innate host defense mechanisms under a biofilm is decreased leading to bacterial persistence. It has also been shown that biofilms promote the establishment and persistence of *K.* *pneumoniae*^9^. Biofilm formation is a significant problem as it renders infections more difficult to control^10^. To date, an alternative approach to novel antimicrobial therapy has been focused on inhibition of the virulence factors of bacterial pathogens. Phages are currently considered as promising antimicrobial agents for biofilm prevention and control^11^.

In light of the problem of the development and spread of antibiotic resistant bacteria, phages have been investigated as alternative antimicrobial agents to treat bacterial infections in humans^12–14^, and several studies have isolated and characterize phages specific for *K.* *pneumoniae*^15–18^. For example *K.* *pneumoniae* phage KN2^19^, NTUH-K2044-K1-1^20^, phage KP36^21^ have exhibited robust activities with high target specificity, possibly as a consequence of their depolymerases and polysaccharide degrading enzymes which can degrade polysaccharides such as capsular polysaccharide (CPS), extracellular polymeric substance (EPS) and biofilm^22^. In animal model studies, a lytic phage φBO1E targeting carbapenemase-producing *K.* *pneumoniae* has been shown to be able to protect infected *Galleria* *mellonella* larvae from death^23^. Treatment with phage 1513 reduced the levels of *K.* *pneumoniae* and improved lesions in mouse lungs^24^, and *K.* *pneumoniae*-mediated respiratory infections in mice could be rescued by phage SS^25^. Moreover, phage φNK5 is a potential therapeutic agent for *K.* *pneumoniae*-induced liver infection in mice^26^.

To develop an effective antimicrobial agent, phage KP1801 infecting ESBL-KP was isolated. Physical characterization, host range, replication kinetics, lytic activity, stability, whole genome sequence, proteome analysis, antibiofilm activities, and in vivo antibacterial activities of phage KP1801 were investigated. In addition, as there have been few proteomic analysis of phages, a shotgun proteome analysis was undertaken to increase our understanding of the protein composition of phage KP1801.

## Results

### Characterization of ESBL-KP

Most of *K.* *pneumoniae* isolates produced ESBL enzyme, classified as ESBL-KP. All isolates were resistant to ampicillin (AMP), 18 (90%) to gentamycin (GEN), 15 (75%) to cefotaxime (CAZ), 17 (85%) to ceftazidime (CTX), and 7 (35%) to ciprofloxacin (CIP). All of them were susceptible to imipenem (IMP) and meropenem (MER) (Supplementary Table S1).

### Pulsed Field Gel Electrophoresis (PFGE)

**Figure 1:**
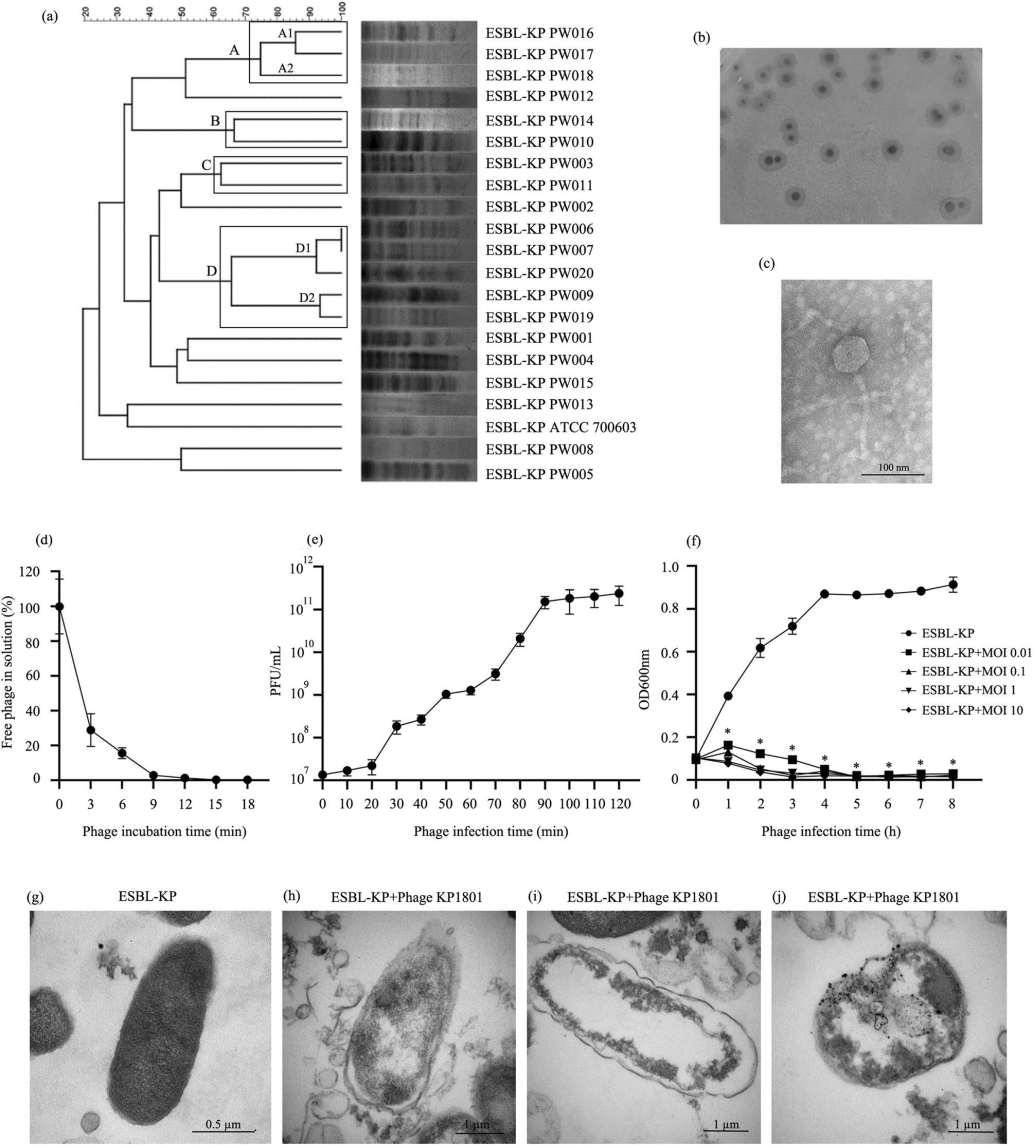
PFGE Genotyping**,** isolation and biological characterization of phage (**a**) Relationships of the 20 ESBL-KP isolates based on PFGE using *XbaI*. The interpretation of the PFGE patterns was performed with BioNumerics software using the Dice similarity coefficient; (**b**) plaque morphology of phage KP1801; (**c**) TEM of phage KP1801; (**d**) adsorption rate; (**e**) one-step growth curve; (**f**) lytic effect of phage KP1801 in vitro; (**g**) TEM of ESBL-KP; (**h**–**j**) TEM of phage KP1801 infected ESBL-KP. Experiments were undertaken independently in duplicate with duplicate assay. The data show the mean ± SD (*, *P* value < 0.05).

Twenty clinical isolates of ESBL-KP were grouped into 4 X*baI*-PFGE main clusters with an 60% similarity value as cutoff point, designated as A, B, C and D (Fig. 1a). Three isolates were categorized into clusters A and divided into 2 sub-clusters (A1 and A2) based on a 74% of similarity value. Cluster B with 2 isolates had more than 65% similarity values. Cluster C had 2 isolates based on a 62% of similarity value. Five isolates were categorized into clusters D and divided into 2 sub-clusters (D1 and D2) based on a 65% of similarity value. Eight isolates were categorized into unrelated strains. ESBL-KP (ATCC 700603) was phylogenetically similar to ESBL-KP PW012 isolates only 33%.

### Phage isolation, purification and physical characterization

A phage was isolated from treated hospital wastewater after enrichment and plaque purification. The isolated phage was designated as phage KP1801. Phage KP1801 produced large clear plaques of 2–7 mm in diameter surrounded by a translucent halo on an ESBL-KP lawn (Fig. 1b). The physical characterization of the phage under TEM revealed that phage KP1801 is non-enveloped, and possess a hexagonal head and a tail. Phages capsid diameter was 53–58 nm, with a long non-contractile tail of approximately 137 nm in length (Fig. 1c). Phage KP1801 showed morphological characteristics similar to that of many other double-stranded (ds) DNA phages. They were classified as being in the family *Siphoviridae*, order *Caudovirales*.

### Phage host range and efficiency of plating (EOP)

The host range of the phage KP1801 was evaluated by a spot test. The ability to produce a lytic zone in 20 clinical ESBL-KP isolates, ESBL-KP (ATCC 700603), *A.baumannii,* *E.* *coli,* MRSA*,* and *P.* *aeruginosa* was determined. Phage KP1801 lysed 50% (10/20) of clinical strains (Supplementary Table S2). The results indicated that phage KP1801 showed broad activity against clinical ESBL-KP isolates.

To further determine the lytic activity of ability of phage KP1801, an EOP assay was performed. No strain showed a higher EOP value than the host strain. High, moderate and low production were found for 5, 3 and 2 isolates, respectively (Supplementary Table S2).

### In vitro characterization of phage

A phage adsorption assay showed that more than 70% of the phage particles were adsorbed to the host cell within 3 min. The percentage of adsorption was nearly 100% after 9 min (Fig. 1d). The results indicated that the adsorption velocity of KP1801 was 3 × 10^5^ PFU/min. The time for phage multiplication in the host, the latency time and the amount of phage released from infected cells, and burst size was determined by a single-step growth curve. The latent time period of phage KP1801 was 35 min and the burst size was estimated to be approximately 300 virions per infected bacterial cell (Fig. 1e). The ESBL-KP lytic activity of phage KP1801 was determined at different MOIs for 8 h in parallel with uninfected ESBL-KP as a control. The results showed that the absorbance of uninfected control ESBL-KP continuously increased, while the absorbance of phage KP1801 infected bacteria at MOI of 0.01, 0.1, 1 and 10 was significantly reduced after 1 h post infection when compared with control. The results showed that phage KP1801 at MOI of 0.1, 1 and 10 significantly decreased the absorbance of ESBL-KP PW006 isolate by 3 h post incubation, and by 5 h at MOI of 0.001 (Fig. 1f).

### Transmission electron microscopy of phage infected bacteria

TEM was used to examine the ultrastructural changes in phage infected bacteria. Uninfected ESBL-KP cells displayed regular morphology with an intact cell membrane (Fig. 1g). For phage KP1801 infected ESBL-KP cells, the phage induced pits in the cell wall. The intracellular density was changed and the cell membrane had disintegrated, leading to ESBL-KP cell damage and eventually completely lysed cells. Large electron-dense granules were observed inside the cells (Fig. 1h, i, j).

### Thermal, pH, UV radiation and long-term stability tests

**Figure 2:**
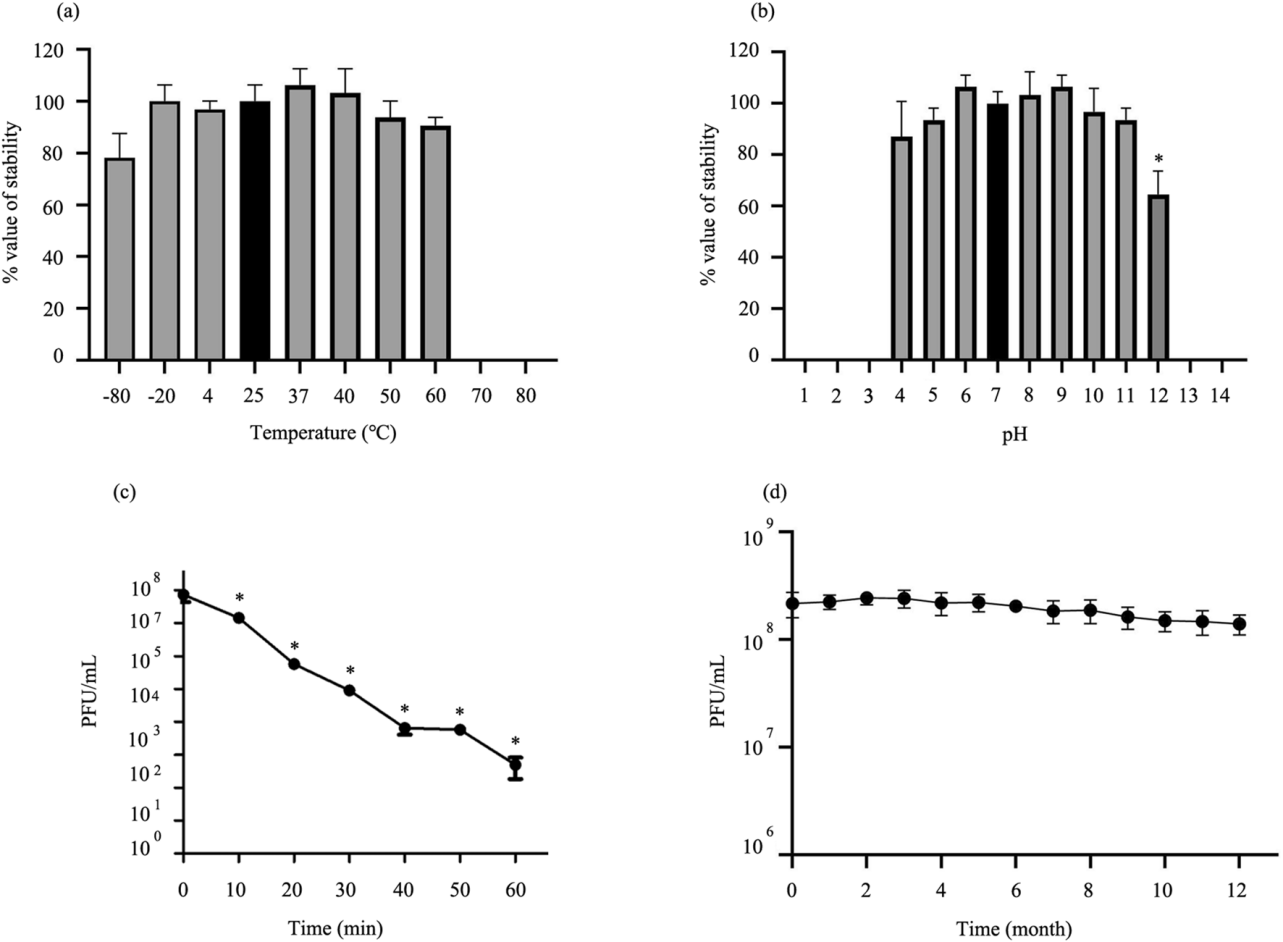
Biological characterization of phage KP1801. (**a**) Effect of temperature on phage KP1801 stability; (**b**) effect of pH on phage KP1801 stability; (**c**) effect of UV on phage KP1801; (**d**) long-term storage of phage KP1801. Experiments were undertaken independently in duplicate with duplicate assay. The data show the mean ± SD (*, *P* value < 0.05 for all treatment groups as compared to control).

Optimal temperature and pH were determined by testing the stability of the phage under varied conditions. The thermal stability of the phage was determined after incubation at − 80 to 80 °C for 2 h. There were no significant changes of phage viability between − 80 to 60 °C, but the phage was completely inactivated by incubation at a temperature higher than 70 °C (Fig. 2a). For phage stability at different pH, phage was incubated in buffers of different pH ranging from 1 to 14 for 2 h. The phage showed no significant variation in stability after incubation at pH 4–11. Incubation at pH 12 significantly reduced phage viability. The phage completely lost viability at pH 1, 2, 3, 13, and 14 (Fig. 2b). The stability and infectivity of phage KP1801 after UV light exposure was tested. Phage titer was significantly reduced by approximately 80% after 10 min UV exposure, and less than 0.0007% viability was observed after 60 min exposure (Fig. 2c). Additionally, the long-term stability of phage KP1801 at 4 °C was also monitored monthly for 12 months. The results showed that the no significant reduction of phage titer over the 12 months, indicating good stability of the phage KP1801 (Fig. 2d).

### Whole genome characterization and analysis

**Figure 3:**
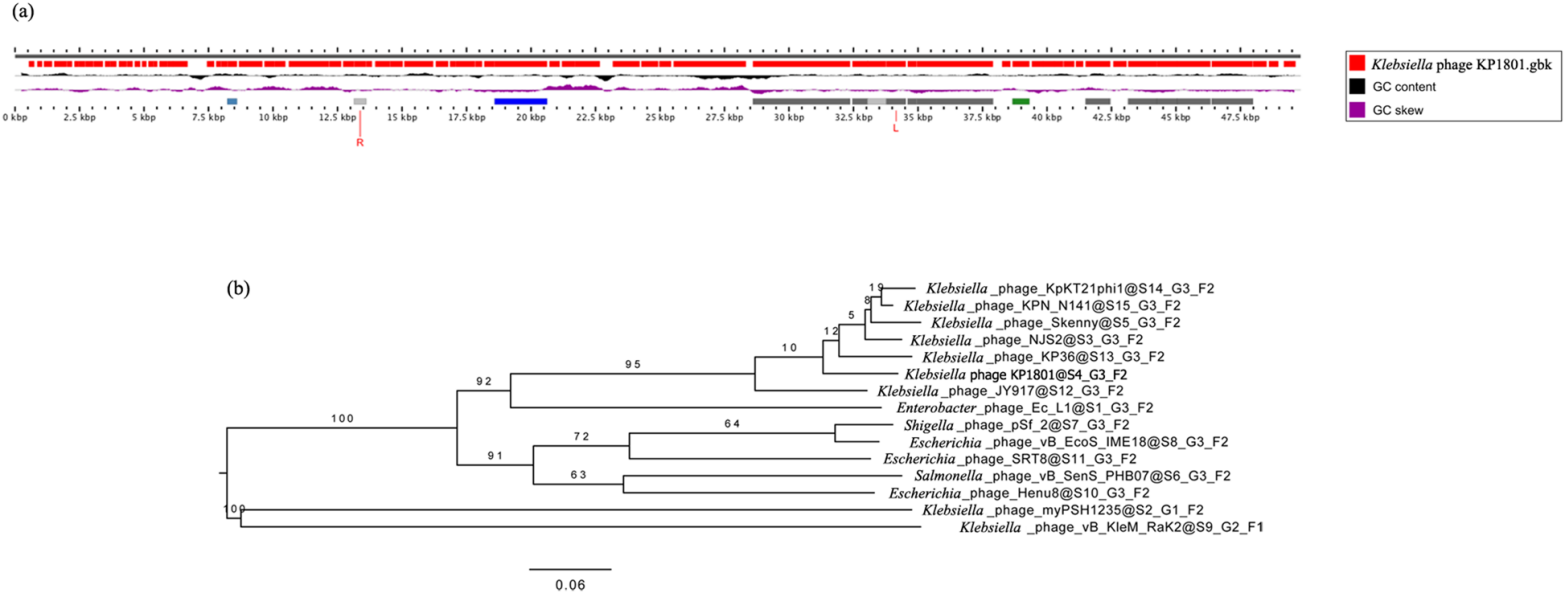
Phage KP1801 whole genome analysis. (**a**) Linear representation of the linear genome; (**b**) whole genome comparison of phage KP1801 and others phage.

The whole genome of phage KP1801 was sequenced, analyzed and deposited in the GenBank database with the accession number MN783016.1. The analysis revealed that the genome of phage KP1801 was a linear double stranded DNA molecule (Fig. 3a). The number of bases in the assembled sequence of phage KP1801 was 49,835 basepairs long, with a GC content of 50.26% and a coding percentage of 89.2%. The genome contained 75 putative open reading frames (ORFs), encoding 75 genes (Supplementary Table S3). A total of 57 ORFs were presented on the negative strand, with 18 ORFs on the positive strand. The majority of the ORFs presented an ATG start codon (97.3%), while 2.7% started with GTG. There were no tRNA, rRNA and tmRNA genes.

To find homologs of the proteins encoded by the predicted ORFs, sequences were searched against the NCBI database. Only 25 ORFs (33.3%) were predicted and determined to encode putative functional proteins, while 50 ORFs (66.7%) were assigned to be hypothetical proteins. A total of 19 ORF-encoded putative functional proteins were present on the negative strand, while 6 ORF-encoded putative functional proteins were present on the positive strand. The biggest and smallest ORF encodes hypothetical proteins with 1,256 and 39 amino acids at ORF52 and ORF73, respectively. The genome of phage encoded proteins for structural (tail fiber protein, tail assembly protein, tail tip protein, minor tail protein, tail length type-measure protein, capsid decoration protein, capsid maturation protease, head morphogenesis protein), host lysis (U-spanin, endolysin, and holin), DNA replication/modification (methyltrasferase type11, DNA adenine methytransferase, DNA helicase, HNH endonuclease, primase, exonuclease, putative single stranded DNA binding protein), packaging (portal protein, terminase large subunit, terminase small subunit), and additional functions (putative phosphoesterase). The sequences were disposed into the *Siphoviridae* head and tail morphogenesis modules and both modules of phage KP1801 showed highest similarity to *Klebsiella* phage KpKT21phi1 (MK278861.1) (87.2% and 76.3%, respectively).

The evolutionary relationship of phage KP1801 genome was compared with other phage genomes by the Genome-BLAST Distance Phylogeny method. Phage KP1801 was most closely related to *Klebsiella* phage KP36 (NC_029099.1). Phage KP1801 was clustered into the same genus with *Klebsiella* phage KP36, phage NJS2 (MH633485.1), phage KPN N141 (MF415412.1), phage KpKT21phi1 and phage Skenny (MK931444.1). (Fig. 3b). All five genomes were assigned to the order *Caudovirales*, *Siphoviridae* family, *Tunavirinae* subfamily. The evolution of this cluster was closed to *Klebsiella* phage JY917 (MG894052.1) and *Enterobacter* phage Ec_L1 (NC_042122.1), respectively. Moreover, phage KP1801 genome was also compared to other phages which infect specific hosts in the order *Caudovirales.* The cluster of *Escherichia* phage vB_EcoS_IME18 (MH051911.1), *Escherichia* phage Henu8 (MN055691.1), *Escherichia* phage SRT8 (NC_042043.1), *Salmonella* phage vB_SenS_PHB07 (MH102284.1), and *Shigella* phage pSf-2 (NC_026010.1) was less related to phage KP1801. There was no related evolutionary relationship between *Klebsiella* phage vB_KleM-RaK2 (NC_019526.1), *Klebsiella* phage myPSH1235 (MG972768.1), and phage KP1801.

The nucleotide homology was also analyzed using the BLASTn tool. Phage KP1801 was highly homologous to phage NJS2 (96.3%), phage KPN N141 (96%), phage Skenny (96%), *Klebsiella* phage JY917 (95.8%), phage KpKT21phi1 (95.6%), *Klebsiella* phage KP36 (95.3%), *Escherichia* phage Henu8 (83.5%), *Enterobacter* phage Ec_L1 (82.2%), *Salmonella* phage vB_SenS_PHB07 (79.5%), *Escherichia* phage vB_EcoS_IME18 (74.5%), *Shigella* phage pSf-2 (74.2%), and *Escherichia* phage SRT8 (72.5%), respectively. There was no homology between *Klebsiella* phage vB_KleM-RaK2, *Klebsiella* phage myPSH1235, and phage KP1801.

**Figure 4:**
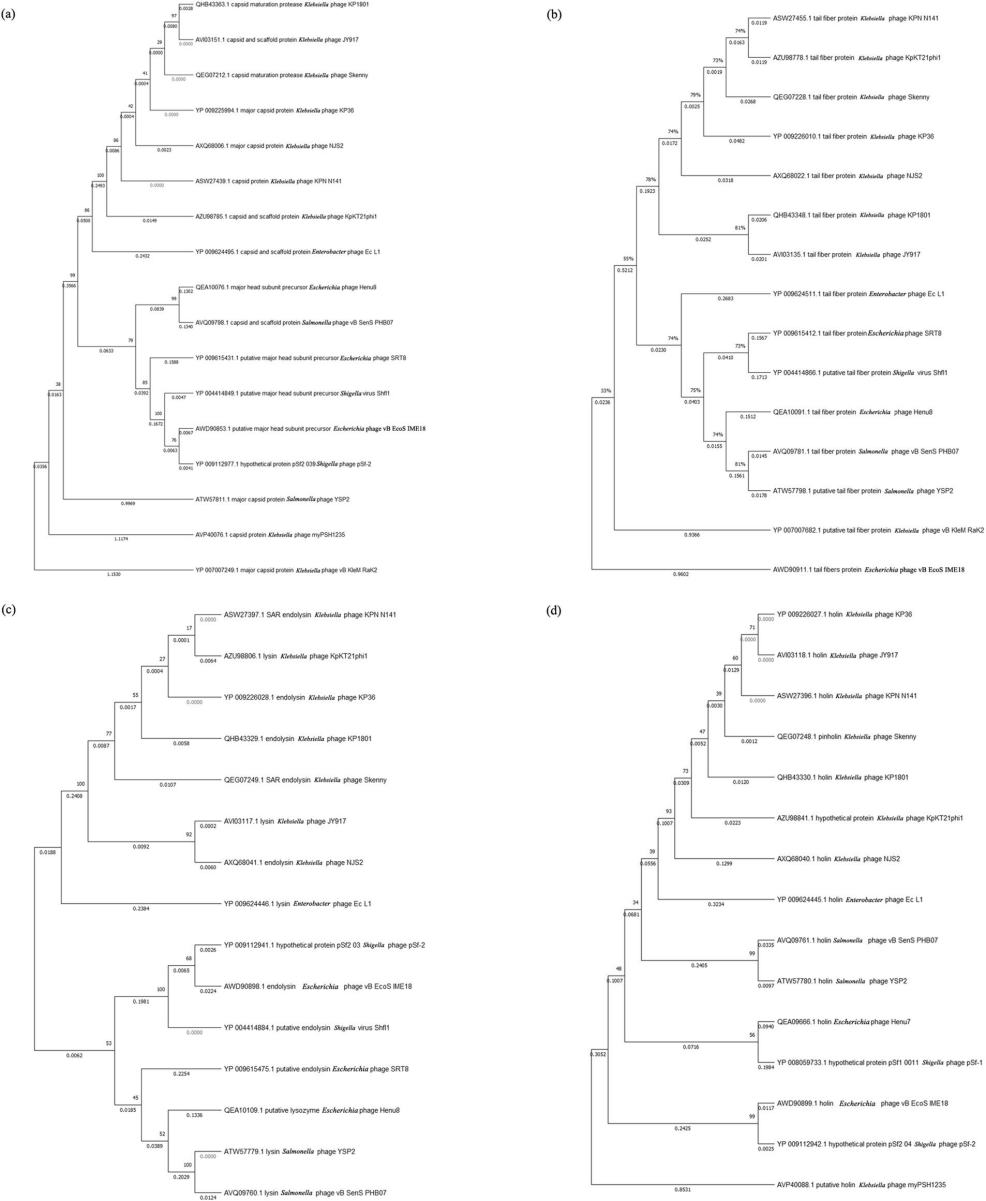
Phylogenetic tree analysis. (**a**) capsid maturation protease; (**b**) tail fiber protein; (**c**) endolysin; (**d**) holin.

To analyze the phage evolutionary relationship, a phylogenetic tree of specific proteins was constructed for comparative analysis using Mega-X and the sequence identity matrix was determined by BioEdit. The sequence homologies of the capsid maturation protease, tail fiber, endolysin, and holin proteins of phage KP1801 were used to determine the relatedness. The phylogenetic tree and sequence identity matrix indicated that capsid maturation protease of phage KP1801 was closely related to *Klebsiella* phage JY917 (99.7% sequence identity matrix) (Fig. 4a). The nucleotide homology of tail fiber protein was similar to *Klebsiella* phage JY917 (96% sequence identity matrix) (Fig. 4b). Endolysin and holin of phage KP1801 were related to *Klebsiella* phage KP36 (99.3% sequence identity matrix) and *Klebsiella* phage Skenny (98.5% sequence identity matrix), respectively (Fig. 4c, d). The sequence homologies of phage KP1801 proteins were also compared to other phages which infect specific host in the order *Caudovirales.* The results showed that the protein sequences were specific in some phages including phage KP1801. The results of the sequence homology analysis confirmed that phage KP1801 had homology to *Webervirus,* subfamily *Tunavirinae*, family *Siphoviridae*, order *Caudovirales*.

### SDS-PAGE and proteomic analysis

The structural proteins of phage KP1801 were analyzed by SDS-PAGE. Phage KP1801 showed a pattern of 5 major protein bands and 15 minor protein bands, with molecular masses ranging from below 15 to around 260 kDa (Supplementary Fig. S1). The proteome of phage KP1801 was analyzed in a total shotgun proteome analysis. The analysis detected 323 proteins, consisting of 234 proteins with known functions and 89 proteins with unknown functions (Supplementary Table S4).

### Phage KP1801 against ESBL-KP biofilm

**Figure 5:**
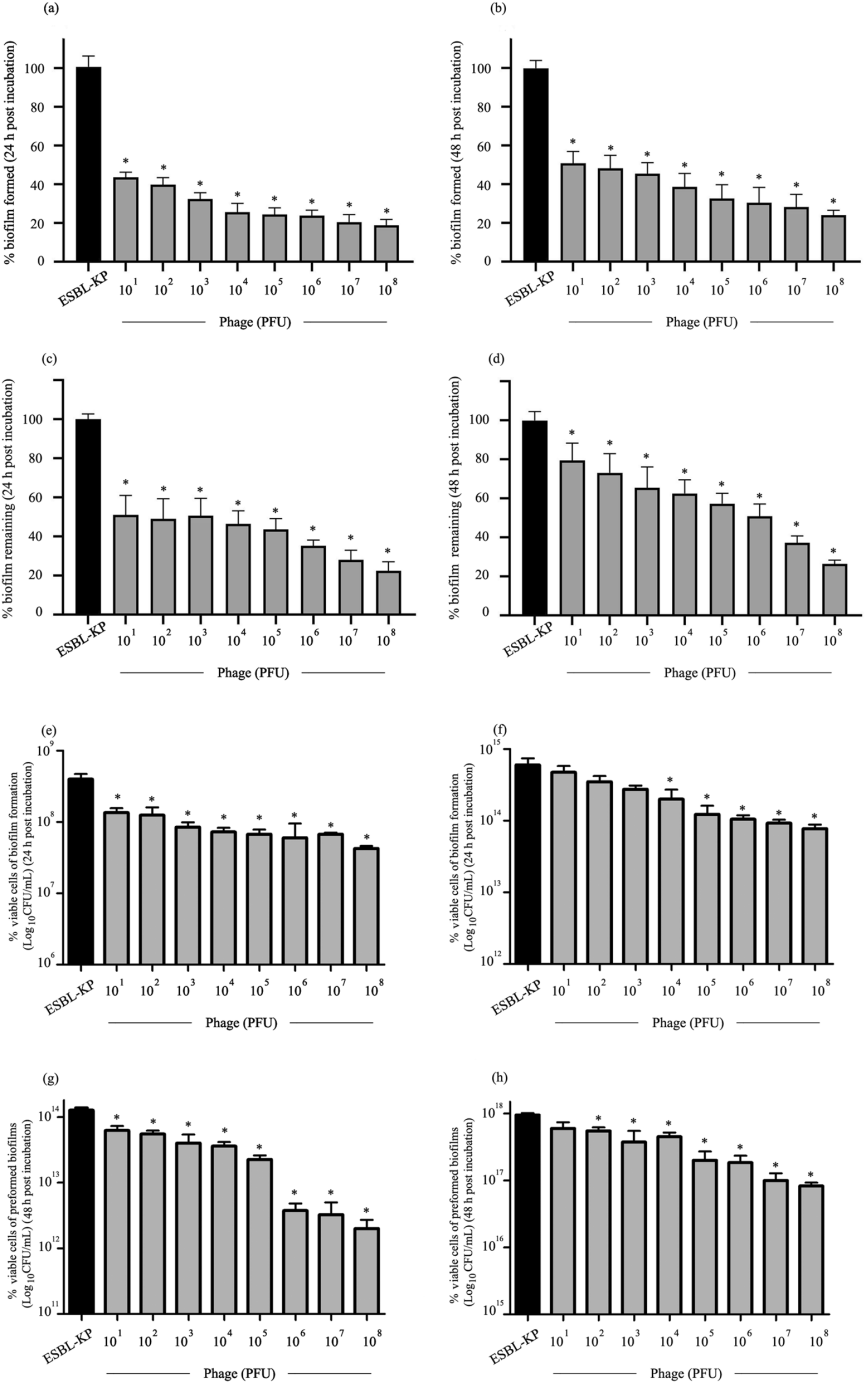
Effect of phage KP1801 on ESBL-KP biofilm. (**a**) Effect of phage on biomass of biofilm formation at 24 h; (**b**) effect of phage on biomass of biofilm formation at 48 h; (**c**) effect of phage on biomass of preformed biofilm at 24 h; (**d**) effect of phage on biomass of preformed biofilm at 48 h. Experiments were undertaken independently in triplicate with duplicate assay; (**e**) Effect of phage on viable cell numbers in biofilm formation experiment at 24 h; (**f**) effect of phage on viable cell numbers of biofilm formation experiment at 48 h; (**g**) effect of phage on viable cell numbers of preformed biofilm experiment at 24 h; (**h**) effect of phage on viable cell numbers of preformed biofilm experiment at 48 h. Experiments were undertaken independently in duplicate with duplicate assay. The data show the mean ± SD (*, *P* value < 0.05).

The activity of phage KP1801 in inhibiting and removing ESBL-KP biofilm was determined by standard crystal violet staining and viable cell counts. For biofilm formation, the biofilm of ESBL-KP was formed in the presence of phage. Phage KP1801 at 10^1^–10^8^ PFU/well significantly inhibited biofilm biomass at 24 and 48 h post incubation from 56 to 81% (*P* < 0.0001) and 49 to 76% (*P* < 0.0001), compared to the negative treatment control, respectively (Fig. 5a, b). For the activity of phage KP1801 against preformed biofilms, biofilms were established for 24 and 48 h, leading to the establishment of mature biofilm followed by treatment with phage. Phage KP1801 at 10^1^–10^8^ PFU/well significantly removed preformed biofilm from 49 to 72% (*P* < 0.0001) and 20 to 74% (*P* ≤ 0.0005) at 24 and 48 h post treatment, respectively (Fig. 5c, d). The results indicated that treatment with phage KP1801 significantly reduced biofilm biomass in both biofilm formation and preformed biofilms, in a dose dependent manner.

The viable cells remaining after the biofilm reduction experiments were determined. Phage KP1801 at 10^1^–10^8^ PFU/well significantly decreased the number of viable cells remaining in the biofilm formation and preformed biofilm experiments at 24 h post incubation (*P* < 0.05) (Fig. 5e, g). No significant reduction of viable cells was seen in the biofilm formation experiment after incubation with 10^1^–10^3^ PFU/well at 48 h post incubation, although a significant reduction was seen after incubation with 10^4^–10^8^ PFU/well (*P* < 0.05) (Fig. 5f). In the preformed biofilm experiment, a significant reduction in cell number was seen after incubation with 10^2^–10^8^ PFU/well (*P* < 0.05) (Fig. 5h).

**Figure 6:**
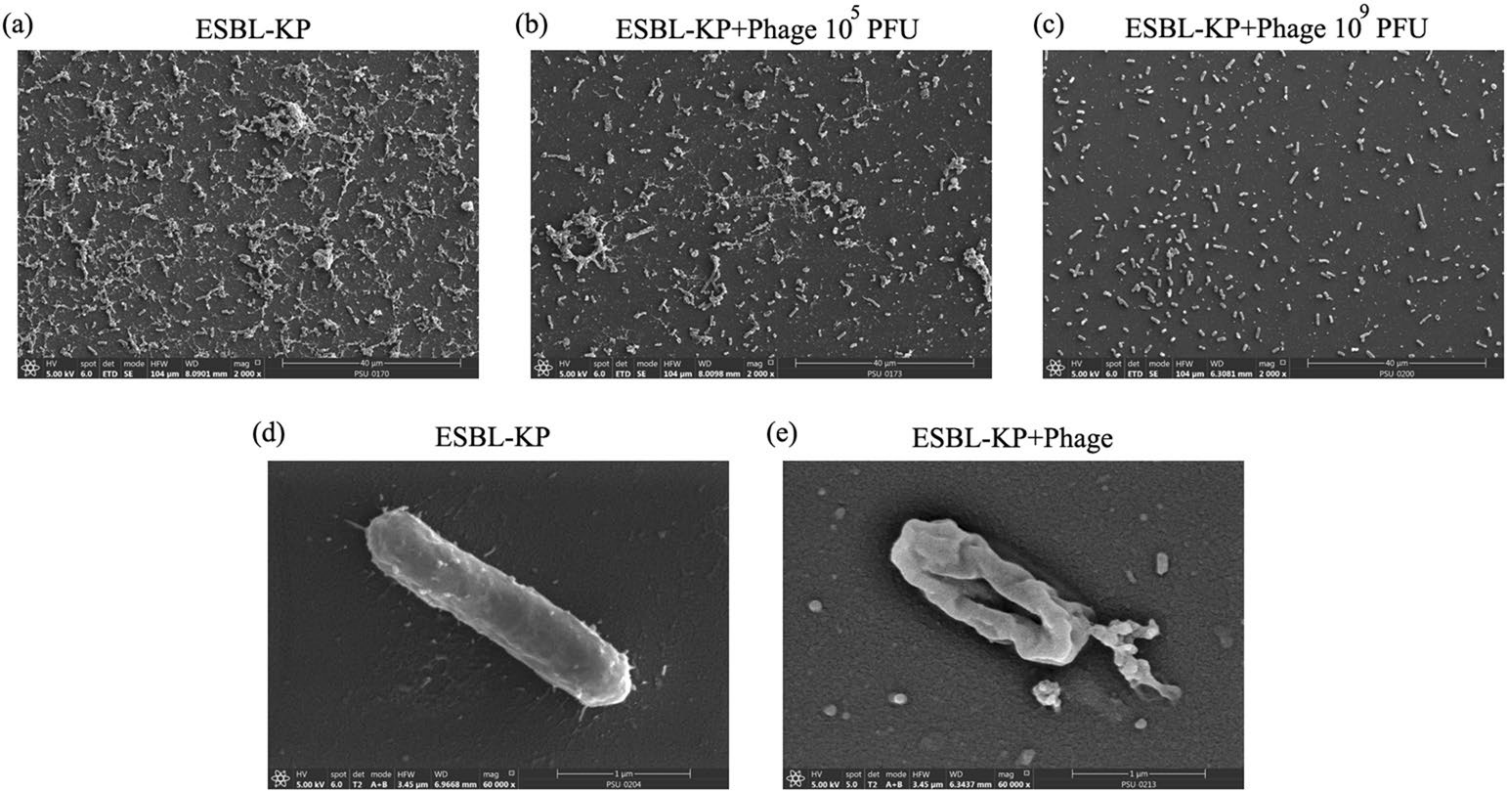
Effect of phage KP1801 on ESBL-KP biofilm. (**a**) ESBL-KP biofilm; (**b**) ESBL-KP biofilm treated with phage at 10^5^ PFU; (**c**) ESBL-KP biofilm treated with phage at 10^9^ PFU. Cellular ultrastructure level of ESBL-KP within biofilm conditions when not treated (**d**) or treated (**e**) with phage under FE-SEM.

SEM analysis of ESBL-KP biofilm confirmed that ESBL-KP was able to form large amounts of biofilm (Fig. 6a). The amount of ESBL-KP biofilm was decreased after treatment with phage KP1801 at 10^5^ and 10^9^ PFU/well (Fig. 6b, c). The cellular ultrastructure of ESBL-KP under biofilm conditions when treated with phage was also examined under SEM. Control untreated ESBL-KP showed a rod-shaped with smooth surfaces (Fig. 6d). ESBL-KP in the presence of phage KP1801 showed a rounded appearances of the cells and membrane shrinkage. Pits on the cell membrane were observed, indicating disruption of the cell wall and cytoplasmic membrane (Fig. 6e).

### In vivo efficiency of phage treatment

The efficacy of phage KP1801 was evaluated in vivo using a *G.* *mellonella* larvae model. To evaluate the virulence of ESBL-KP, the melanization and survival rate of larvae after the bacterial infection was observed and LD50 was then calculated. ESBL-KP in the range of 5 × 10^4^ to 5 × 10^7^ CFU/mL (20 µL per larvae) was tested. Increased melanization of larvae after the inoculation with ESBL-KP was observed. At day 5 post infection, larvae in uninfected and PBS injected control groups were healthy and there was no melanization and mortality was in negative control groups. The mortality of larvae increased in a dose dependent manner, and ESBL-KP at 4 × 10^6^ CFU/mL was determined to be the LD50, with 50% larvae dead within 5 days (Supplementary Fig. S2).

**Figure 7:**
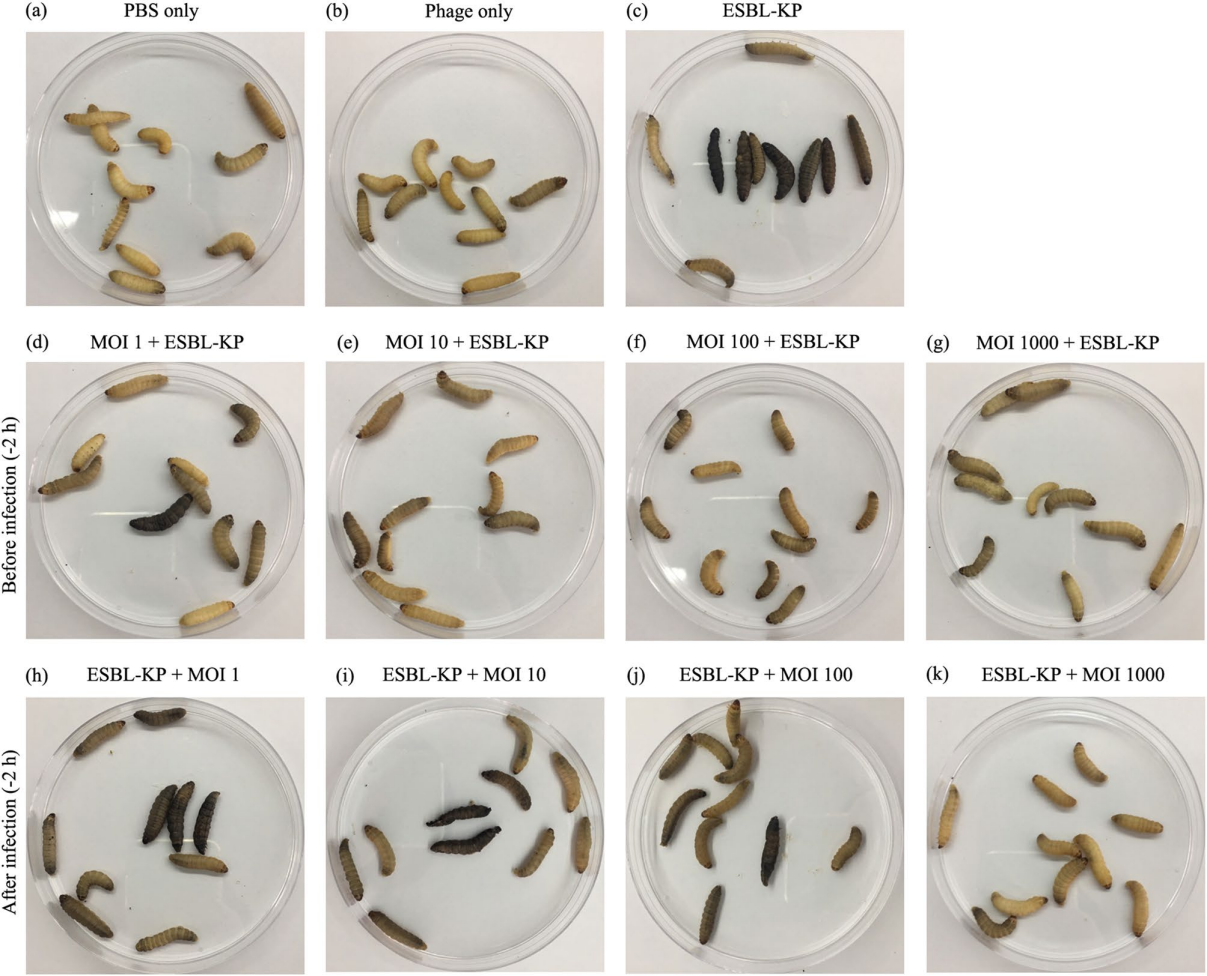
Morphology of *G.* *mellonella* after phage KP1801 treatment. If the larvae moved, responded to physical stimuli and showed no sign of melanization, it was considered alive. If there was no movement or the larvae was melanized, it was considered as dead.

**Figure 8:**
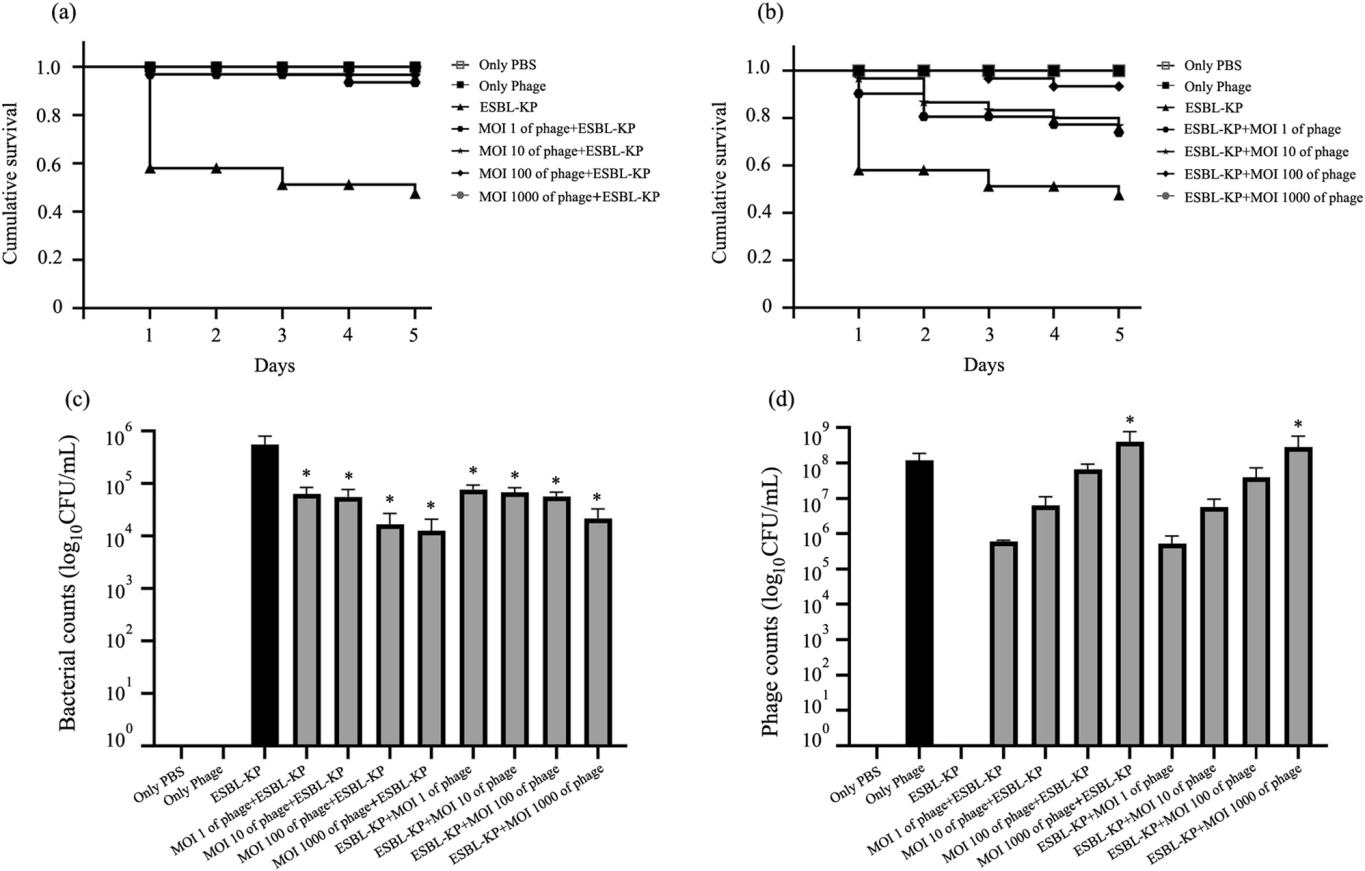
Anti-ESBL-KP activity of phage KP1801 on *G.* *mellonella.* (**a**) Survival rate on prophylactic treatment; (**b**) survival rate on therapeutic treatment; (**c**) bacteria count in *G.* *mellonella* at day 5 post infection; (**e**) phage titre in *G.* *mellonella* at day 5 post infection. Experiments were undertaken independently in triplicate with 10 larvae per group. The data show the mean ± SD (*, *P* value < 0.05).

For the treatment experiments, *G.* *mellonella* larvae were injected with a single phage dose containing the LD50 dose of ESBL-KP. In addition, larvae were injected with only PBS and the highest phage dose (4 × 10^9^ PFU/mL). The larvae were observed and melanization and the survival rate was recorded for 5 days post inoculation. Both bacteria and phage were recovered from viable larvae at the end of the observation period. Injection of either PBS or phage only did not cause any melanization or the death of any larvae (Fig. 7a, b). To evaluate the phage in a prophylactic model, the larvae were inoculated with phage KP1801 for 2 h followed by infection with ESBL-KP. Control larvae injected with an LD50 dose became melanized (Fig. 7c) and the mortality rate was approximately 50% as compared to negative controls (Fig. 8a, b). One hundred percent of survival rate in phage treatment groups at MOI of 10 and 100 was observed up to 2 and 3 days, respectively. At day 5 post infection, melanization and death were observed in all of the groups except for the phage treatment group at MOI of 1,000 (Fig. 7d–g), in which approximately 47% of infected larvae survived after ESBL-KP infection. The survival rate of larvae was 93, 97, 97, and 100% when pre-treating with phage with MOI of 1, 10, 100, and 1,000, respectively. Phage KP1801 significantly reduced the mortality of larvae when compared with a positive control (*P* ≤ 0.0029) (Fig. 8a). To determine the bacterial load after treatment, the bacteria in larvae were counted. The bacterial load was significantly decreased by approximately 74, 77, 81, and 89% at MOI of 1, 10, 100, and 1,000, respectively (*P* < 0.0001) (Fig. 8c). For the therapeutic model, the larvae were infected with ESBL-KP 2 h prior to their injection with phage KP1801. The results showed that 100% survival rate in phage treatment groups at MOI of 100 and 1,000 was observed up to 2 and 5 days, respectively. At day 5 post treatment, melanization and mortality of larvae occurred in all of the groups (Fig. 7h–j) except for the phage treatment group at MOI of 1,000 (Fig. 7k). A single dose of phage at MOI of 1, 10, 100, and 1,000 was effective with a survival rate of 73, 77, 93, and 100%. A statistically significant difference between the survival curves of infected larvae and infected larvae treated with phage was seen (*P* ≤ 0.0249) (Fig. 8b). The bacterial load was significantly decreased by 79, 81, 94, and 96% at MOI of 1, 10, MOI of 100, and MOI of 1,000, respectively (*P* < 0.0001) (Fig. 8c). For phage replication, the number of phage in larvae significantly increased approximately 1log at MOI of 1,000 in both prophylactic (*P* = 0.001) and treatment (*P* = 0.0133) models (Fig. 8d).

## Discussion

ESBL-KP, an opportunistic pathogen associated with nosocomial infections has been reported as the cause of an increasing number of community-acquired disease and hospital-acquired infections, and is associated with significant morbidity and mortality^27^. ESBL-KP which is susceptible to a limited range of effective antibiotics has become an important pathogen and is a first priority antibiotic-resistant bacteria requiring the development of new antibiotics^7^. This study characterized a new phage, KP1801 followed by determination of the antimicrobial activity of phage in vitro and in vivo.

Phage KP1801 with a broad isolate range, formed large plaques surrounded by a translucent halo on bacterial lawns, indicating the presence of phage tail related exopolysaccharide (EPS) depolymerases, enzymes with EPS-degrading activity which are typically located on the phage tail, and endolysin, an enzyme related to host cell degradation^28^.

Based on morphologic characteristics, phage KP1801 was composed of an isometric head with a noncontractile tail, so the phage was classified as belonging to the *Siphoviridae* family, in the order *Caudoviridae*. Other members of the *Kp36* *virus* genus, *Siphoviridae* family-specific for *K.* *pneumoniae* display an isomeric head of 45–80 nm diameter and a long non-contractile tail of 90–190 nm long^15,24,29,30^. Phage KP1801 showed a high adsorption rate with high antibacterial activity. Phage KP1801 has a short time multiplication time within the host, with a large amount of phage released from infected cells so phage KP1801 therefore has the potential to be used as a bacterial treatment tool.

The main influences on the application of phage are external environment factors. The pH and temperature of phage storage are the important factors, affecting phage activity and stability. No significant changes in phage KP1801 viability were observed after incubation at temperatures in the range − 80 to 60 °C, and over a pH range of pH 4–12. For phage tolerance, the viability of phage is related to its structure, and the tailed phage *Siphoviridae,* have been reported as the most stable phage in adverse conditions^31,32^. Under long term storage phage KP1801, viability was not affected by long-term storage of up to a year. Furthermore, the integrity of phage under UV radiation, the most damaging environmental factor was also investigated, and phage KP1801 was less susceptible to UV radiation as compared with phage BF25/12, a member of *Podoviridae* family^33^. Phage KP1801 was also shown to be highly effective at lysing ESBL-KP in vitro, supporting the possibility of using this phage as a potential antibacterial therapeutic agent.

Genomic analysis showed that phage KP1801 is a newly discovered phage, which is most closely related to the *Siphoviridae* phage KP36. KP36 is placed in the *Kp36* *virus* genus (*Webervirus*) of the subfamily *Tunavirinae*, family *Siphoviridae*, order *Caudovirales*. While the number of ORFs and the genome size of phage KP1801 are quite close to phage TSK1^15^, the percentage of identity for these genome sequences was only 41.6%. Several functional putative proteins from phage KP1801 were identified and more than 50% of predicted phage KP1801 proteins are hypothetical proteins whose functions are unknown. According to previous reports, depolymerases can be found in the tail fiber protein or the tail spike protein. Tail fiber proteins of *Klebsiella* phage JY917, *Klebsiella* phage KP36, and *Klebsiella* phage Sushi (AKQ07492.1) have been reported to possess capsule depolymerase activity^21,34^, and the tail fiber protein encoded by KP1801 ORF52 might possess capsule depolymerase activity as this ORF shared amino acid sequence homology to tail fiber proteins of other *Klebsiella* phages which possess capsule depolymerases activity such as *Klebsiella* phage JY917 (96%), *Klebsiella* phage KP36 (86.2%) and *Klebsiella* phage Sushi (77.7%). Three important proteins related to antibacterial activity were identified including the tail fiber, a protein displaying capsule depolymerase activities that can disrupt biofilm potential, spanin, a phage lysis protein that disrupts the outer membrane of Gram-negative bacteria, and holin, an enzyme for host cell disruption^34,35^.

The genetic relationship between phage KP1801 and other phages was studied by comparison of specific phage proteins including the capsid maturation protease, tail fiber protein, endolysin and holin. A phylogenetic analysis of phage KP1801 capsid maturation protease showed that KP1801 was more similar to *Klebsiella* phage JY917 than *Klebsiella* phage NJS2. Similarly, genomic sequence comparison of the tail fiber protein showed that tail fiber protein of phage KP1801 was most closely related *Klebsiella* phage JY917.

Endolysin and holin are essential proteins involved in host cell lysis. Endolysin, has shown potent antimicrobial activity against planktonic bacteria and bacteria in biofilm. While endolysin is more effective against Gram-positive bacteria than Gram-negative bacteria, several recombinant endolysin proteins have been investigated against Gram-negative bacteria. Endolysin LysPA26 inhibited *P.* *aeruginosa* in biofilm formation and killed other Gram-negative bacteria including *K.* *pneumoniae*^36^. Endolysin K11gp3.5 and K32gp15 of *K.* *pneumonia* showed antibacterial activity against *P.* *aeruginosa*^37^. The phylogenetic tree of endolysin showed that endolysin of phage KP1801 was similar to that of *Klebsiella* phage KP36.

Holin, a small membrane protein accumulating in the membrane is a protein clock of phage infection that regulates the timing of the phage infection cycles and mediates host lysis at a specific time point^38^. Holin shares some common characteristics among phages but their sequences are different^39^. A phylogenetic analysis of phage KP1801 holin showed that it was mostly closely related to *Klebsiella* phage Skenny. Taken together, analysis of all selected protein markers supports that phage KP1801 is a member of the family *Siphoviridae.*

There were no lysogen-formation gene clusters such as the CI and CIII genes in the phage genome, indicating that phage KP1801 was a virulent phage in the lytic cycle^40^. Based on the functional putative genes, there were no toxin, virulence or antibiotic-resistant related genes in the phage genome, suggesting that phage KP1801 might be safe for humans and biocontrol. However, more than 50% of phage KP1801 protein have hypothetical functions, and so further study is required to evaluate the safety of the phage.

Several reports have investigated the structural protein proteome, but there are few studies on total protein profiles of phage. Previously 2D electrophoresis coupled with MALDI-TOF was used to investigate the total proteins of a *Myoviridae* phage specific for *Mannheimia* *haemolytica*^41^, while a total of 346 proteins of phage BPA43 specific for ESBL-KP were characterized by shotgun proteomics^42^. In this study shotgun proteomic analysis combined with bioinformatics analysis of phage KP1801 identified 323 proteins. Proteins identified by proteomics were related to proteins found in the genome, leading to the classification of proteins into 6 groups including structural proteins, host infection-related proteins (host lysis), DNA replication/modification-related proteins, packaging proteins, other functions-related proteins, and uncharacterized proteins. Interestingly, some important proteins were detected only in the proteomic analysis such as a depolymerase protein and peptidoglycan hydrolase, a peptidoglycan degrading enzyme^43^. The homology of phage KP1801 proteins with other phages was characterized by searching peptide in UniProt database. Two hundred ninety proteins specifically belonged to phages in the order *Caudovirales,* while 32 proteins belonged to phages in the other orders such as *Bullavirinae,* *Inoviridae,* *Microviridae,* *Riboviria,* and *Tectiviridae.* Moreover, one protein matched to unclassified viruses.

The results of this study have shown that proteome has a greater number of proteins than proteins predicted to be encoded by the phage KP1801 genome. However, the relationship between genomes and their information is still unclear^44^. One open reading frame (ORF) might undergo alternative spicing, a major source of cellular protein diversity, leading to translation into many proteins. Moreover, short open reading frames (sORF) can be possibly missed in genome annotation as a consequence of their small size^45,46^.

*Klebsiella* *pneumoniae* has a high ability to form biofilm, and accumulate bacteria in the early stages of infection^47^. Biofilms are difficult to eradicate by the host immune defenses and antimicrobial agents, increasing the severity of *K.* *pneumoniae* infection^10,48^. Microbial growth in biofilms results in increased tolerance to antibiotics, and for these bacteria antibiotic treatment concentration may need to be increased to 10–1,000 times greater than needed for planktonic bacteria. Moreover, some antibiotics cannot penetrate biofilms to kill bacteria^49^. However, as a result of the exopolysaccharide (EPS) depolymerase activity associated with the phage tail, phage encoding EPS depolymerases can degrade the capsular polysaccharide. Depolymerase also show activity in degrading EPS and facilitating the entry of phage to bacterial cells within a biofilm^50^. This activity is an advantage of phages as compared to antibiotics in killing biofilms^51^. Phage encoding depolymerases have been reported as phage with higher activity against biofilms^52,53^. A number of phage specific to *K*. *pneumoniae* have been isolated, but only some have been investigated for antibiofilm activity. Phage ZCKP1 at an MOI of 50 reduced more than 50% biofilm biomass of MDR *K.* *pneumoniae*^54^, and bacterial biofilm and biofilm formation were also reduced by phage TSK1 against *K*. *pneumoniae*^15^. In this study, a depolymerase protein was detected in the proteomic study. Phage KP1801 inhibited biofilm formation and eradicated preformed biofilm in a dose-dependent manner, highlighting the possibility of using phage KP1801 as a biocontrol agent.

According to a previous report, a combination treatment between phage B5055 and ciprofloxacin killed *K.* *pneumoniae*^55^*.* Phage B5055 has also demonstrated a synergistic ability to eradicate *K.* *pneumoniae* biofilm with beta-lactams^56^. The combination also reduced the development of phage-resistant variants. It is likely that the phage activity in disrupting the bacterial biofilm may allow the diffusion of antibiotics to kill bacteria. In this respect phage therapy has attracted growing interest as an efficacious and alternative therapeutic modality to control biofilm-related infections. It is possible therefore that a combination of phage KP1801 and antibiotics might result in synergy.

*Galleria* *mellonella* has been widely used as an alternative model^57,58^. Using larvae has several advantages over other models including a short lifespan, and larvae are cheap and ethics-free as compared to other systems^59^. *G.* *mellonella* was used as an in vivo model to evaluate the therapeutic efficacy of phage KP1801. Phage KP1801 was not toxic for larvae, and single phage dose showed significant efficacy in prophylactic and therapeutic treatments. In addition, the number of phage in vivo was significantly increased, indicating that phage KP1801 could replicate at the site of infection. However, multiple phage doses might increase the survival rate of larvae, and a phage cocktail could increase further the efficiency of phage therapy. Although *G.* *mellonella* have an innate immunity system as do vertebrates, the larvae do not possess an adaptive immunity system^60^. Further evaluation of phage efficacy in a mammalian animal model system will be useful in the future.

In conclusion, a novel phage, KP1801 with activity against ESBL-KP was isolated and characterized. The phage was classified as being in the order *Caudoviridae*, belonging to the *Siphoviridae* family. The phage exhibited good tolerance to a range of pH and temperature, and was stable under long-term storage conditions. The phage was efficacious in both biofilm inhibition and removal, and showed high efficacy in a *G.* *mellanella* in vivo model. Therapeutic trails will be used to confirm its potential as antibacterial therapeutic agent.

## Material and methods

### Bacterial strains and growth conditions

Twenty clinical isolates of ESBL-KP were isolated from Songklanagarind Hospital, Songkhla Province, Thailand. *Acinetobacter* *baumanii*, *Escherichia* *coli,* methicillin-resistant *Staphylococcus* *aureus* (MRSA), and *Pseudomonas* *aeruginosa* clinical isolates were obtained from the same source. Tryptic soy agar (TSA; Becton, Dickinson and Company, Franklin Lakes, NJ) was used to grow all bacterial isolates overnight at 37 °C. Five colonies on TSA were transferred to 3 mL of sterile tryptic soy broth (TSB; Becton, Dickinson and Company, Franklin Lakes, NJ) and then incubated at 37 °C with continuous shaking at 150 rpm.

### Antimicrobial susceptibility testing and determination of minimum inhibitory concentration (MIC)

Antibiotic susceptibility and MIC were determined in a routine clinical laboratory service laboratory according to the CLSI guidelines^61^. Antibiotic susceptibility of ESBL-KP isolates to ampicillin (AMP) (10 µg), cefotaxime (CAZ) (30 µg), ceftazidime (CTX) (30 µg), ciprofloxacin (CIP) (5 µg), gentamicin (GEN) (10 µg), imipenem (IMP) (10 µg), and meropenem (MER) (10 µg) were determined by the disc diffusion technique. MICs were quantified using E-tests for 3 antibiotics (CAZ, CTX, and CIP). *K.* *pneumoniae* (ATCC 700603) was used as the control.

### Combination disk test

ESBL production was screened by a combination disk test using cefotaxime (30 µg) and ceftazidime (30 µg) discs alone and in combination with clavulanic acid (10 µg) discs^62,63^. *K.* *pneumoniae* (ATCC 700603) was used as the control.

### Pulsed-field gel electrophoresis (PFGE)

The genetics relatedness of the bacterial isolates was determined by PFGE on a CHEF system (Bio-Rad Laboratories, Hercules, CA) using X*ba* I (Thermo Fisher Scientific, Waltham, MA) as a restriction enzyme^64,65^. Briefly, ESBL-KP colonies were inoculated in cell suspension buffer (1 M Tris pH 8, 0.5 M EDTA pH 8, 1 M NaCl) and then further adjusted to give a final OD600 of 1.0. The cell suspension was warmed for 10 min at 55 °C followed by the addition of 20 µL of protenase K (20 mg/ml) and 500 µL of 1.2% low melting agarose prepared in TE buffer. This mixture was quickly dispensed into the plug mold. After 30 min incubation at room temperature, plugs were then incubated for 30 min at 4 °C. Plugs were placed into a tube of 5 ml lysis buffer (1 M Tris-HCl pH 8, 0.5 M EDTA pH 8, 10% SDS) followed by the addition of 25 µL of protenase K. Plugs were incubated for 2 h at 55 °C and lysis buffer was then discarded. After washing with pre-warmed sterile distilled water, plugs were washed with TE buffer, pH 8 and then stored overnight at 4 °C. Plugs were cut into a slice of 3 × 10 mm and then equilibrated with 1X FastDigest buffer for 1 h at 37 °C. The buffer was discarded and plugs were digested with 100 µL of enzyme mixture (10X FastDigest buffer, X*ba* I FastDigest, water). After incubation for 2 h at 37 °C, X*ba* I digested genomic DNA was electrophoresed in 1% agarose gel in 0.5X TBE buffer using a CHEF system (Bio-Rad Laboratories, Inc, Hercules, CA, USA) for 19 h at 6 V/cm, angle 120, temperature 10 °C, initial switch time 2.2 s, and final switch time 54.2 s. The CHEF DNA size standard (1703635, Bio-Rad Laboratories, Inc, CA) was used a molecular reference marker. The gel was stained with ethidium bromide solution and DNA visualized under UV light. DNA banding patterns were analyzed using the software BioNumerics, version 7 (Applied Maths NV, Sint-Martens-Latem, Belgium). A Dice similarity coefficient was employed to compare the obtained PFGE fingerprints.

### Phage enrichment and isolation

A water sample after hospital wastewater treatment was collected from Phattalung hospital, Phattalung Province, Thailand. Based on the antimicrobial susceptibility, ESBL-KP PW006 was used as a host strain for phage isolation. The water sample was centrifuged at 6,400×*g* for 15 min at 4 °C. The supernatant was collected and then filtered through a sterile 0.22 μm filter (Merck Millipore, Burlington, MA) to remove debris and bacterial cells. Ten milliliters of water sample was mixed with 10 ml of TSB followed by inoculation of 200 µL of overnight grown host strain. After incubation overnight at 37 °C with shaking at 150 rpm, the culture was centrifuged at 6,400×*g* for 15 min at 4 °C and the supernatant was filtered through a sterile 0.22 μm filter. A double-layer agar overlay plaque assay was used to detect the isolated phage.

### Phage purification and amplification

A plaque was extracted from the agar overlay with a sterile tip and then re-suspended in 500 μL sterile SM buffer (0.1 M NaCl, 8 mM MgSO_4_, 7H_2_O, 50 mM Tris-HCl pH 7.5) followed by incubation overnight at 4 °C. A tenfold dilution series of lysate was prepared in SM buffer and then used to inoculate TSB containing 200 μL of log-phase of a clinical isolate of ESBL-KP followed by incubation for 15 min. The incubated mixture was supplemented with top agar and then quickly poured onto a TSA plate. The plate was incubated overnight at 37 °C. The single plaque isolation, elution, and re-plating were repeated for five rounds to obtain purified plaques. For phage amplification, double-layer agar overlay plaque assay was performed and phage was eluted from confluent plates by addition of SM buffer and incubation of the plates at 4 °C overnight. The supernatant was collected, pooled and subsequently centrifuged at 6,400×*g* for 20 min at 4 °C. The supernatant was filtered through a 0.22-μm pore filter before addition of one microliter of chloroform per milliliter of phage solution. The phage stock was kept at 4 °C until used.

### Phage concentration

Phage was concentrated as described previously^66^. Briefly, ESBL-KP was infected with phage at a multiplicity of infection (MOI) of 1 and then incubated at 37 °C for 6 h. The mixture was centrifuged to remove host cell debris at 6,000×*g* for 20 min at 4 °C. The supernatant was collected and then filtered through a 0.22-μm pore filter. Phage particles were precipitated with 1 M NaCl and 10% polyethylene glycol 6,000 (PEG6000) (Sigma-Aldrich, St. Louis, MO) at 4 °C, overnight. The phage particles were pelleted by centrifugation at 11,000×*g* for 30 min at 4 °C followed by discarding the supernatant. Phage particles were re-suspended in SM buffer for DNA and protein extractions, while phage particles were re-suspended in PBS buffer for in vivo studies.

### Determination of phage titer

Phage titration was performed as described elsewhere^67,68^. Briefly, a tenfold dilution series of phage were mixed with log-phase ESBL-KP culture and the mixture was supplemented with top TSB soft agar followed by pouring onto a TSB agar plate. Following overnight incubation at 37 °C, the number of plaques was counted. Phage titer was determined as plaque forming unit per mL (PFU/mL).

### Examination of phage morphology by transmission electron microscopy (TEM)

The phage morphology was observed by TEM using a negative staining technique as described elsewhere^66^. Briefly, phage in SM buffer was spotted onto a copper grid and then staining with 2% (vol/vol) uranyl acetate (pH 6.7). The morphology of phage KP1801 was examined under a JEOL JEM-2010 transmission electron microscope at an acceleration voltage of 80 kV.

### Host range analysis

The host range of the obtained phage was determined against 20 clinical isolates of ESBL-KP*,* *K.* *pneumoniae* (ATCC 700603), *A.* *baumannii*, *E.* *coli*, MRSA*,* and *P.* *aeruginosa* using the standard spot test^69^. Briefly, two hundred microliters of each log-phase bacterial isolate was mixed with semi-solid medium and poured onto a TSA plate. Ten microliters of the phage lysate (10^5^ PFU/mL) was dropped onto the overlaid top agar against bacterial strains. Plates were dried and incubated overnight at 37 °C. The presence of lytic zone was observed on the bacterial lawn. The experiment was undertaken as independent duplicates.

### Efficiency of plating (EOP)

The lysis ability of phage was quantitated by the double agar overlay method. EOP was conducted as previously described^70^. Briefly, a series of tenfold dilutions of phage was prepared in SM buffer. Each log-phase bacterial isolate was adjusted to a final OD600 of 0.1 and 200 µL of ESBL-KP was then mixed with 100 µL of phage (MOI = 0.01). The mixtures were incubated for 15 min followed by a double agar overlay. The EOP was calculated as the ratio of the number of lytic plaques produced on the bacterial lawn of the test bacterium to the number of plaques produced on the lawn of the host bacterium. EOP was classified as high production at a ratio ≥ 0.5, moderate production at a ratio 0.1 ≤ EOP < 0.5, low production at a ratio 0.001 < EOP < 0.1 and no production at a ratio ≤ 0.001 The experiment was undertaken independently in duplicate with duplicate plaque assay.

### Phage adsorption rate assay

The adsorption assay was carried out as described previously^66^. A log phase culture of ESBL-KP was incubated with phage KP1801 at MOI of 1 followed by incubation at 37 °C with constant shaking. Samples were collected every 3 min post incubation for 18 min. After centrifugation at 12,000×*g* for 5 min at 4 °C, supernatants were then filtered through a 0.22 μm pore filter. The filtrate was plated by a double layer plaque assay for plaque counting. The experiments were undertaken independently in duplicate with duplicate plaque assay.

### One step growth curve

A one step growth was undertaken as described elsewhere^66^. Briefly, a log-phase culture of ESBL-KP was adjusted to a final OD600 of 0.1 (approximately 1 × 10^8^ CFU/mL). Bacterial suspensions were centrifuged at 6,000×*g* for 20 min at 4 °C. The pellet was resuspended in TSB and then mixed with phage KP1801 at an MOI of 1 followed by incubation for 15 min. The mixture was centrifuged to remove the unbound phage particles at 6,000×*g* for 20 min at 4 °C. The supernatant was discarded and the pellet was then resuspended in TSB followed by incubation at 37 °C. Samples were taken at 10 min intervals until 120 min. Samples were diluted, and then titrated by the soft-agar overlay method. Burst size was calculated as the ratio of the final count of phage particles to the initial count of infected bacterial cells. The experiments were undertaken independently in duplicate with duplicate plaque assay.

### Bacterial cell killing assay

The bacteriolytic efficacy of phage KP1801 was determined. ESBL-KP was infected with phage at MOI of 0.01, 0.1, 1 and 10. Uninfected ESBL-KP was used as a control sample. The mixtures were incubated at 37 °C with shaking and then collected for OD600nm measurement every hour for 8 h. The experiments were undertaken independently in duplicate with duplicate assay.

### TEM of phage-infected bacteria

The effects of phage KP1801 on ESBL-KP cell ultrastructure was visualized by TEM. Briefly, ESBL-KP was infected with phage KP1801 at MOI of 1 for 2 h. The samples were pelleted by centrifugation at 12,000×*g* for 5 min at 4 °C and washed two times with 0.1 M PBS (pH 7.4). After fixation in 2.5% glutaraldehyde/PBS overnight at 4 °C, the samples were washed with PBS and then incubated with 1% (w/v) osmium tetroxide prepared in PBS for 2 h. The fixed samples were dehydrated in a series of ice-cold ethanol washes and then infiltrated by Jembed 812 resin. Thin sections were cut using an ultramicrotome, mounted on nickel grids, followed by staining with uranyl acetate and lead citrate. Micrographs were taken with a JEOL JEM-2010 transmission electron microscope at an acceleration voltage of 100 kV.

### Assessment of phage thermal and pH stability

The effect of different temperatures (− 80 °C, − 20 °C, 4 °C, 25 °C, 37 °C, 40 °C, 50 °C, 60 °C, 70 °C and 80 °C) and pH (1–14) on the stability of phage KP1801 was determined. Briefly, phage lysate was diluted to a final concentration of 10^8^ PFU/mL in a final volume of 1 mL of SM buffer and then incubated at the specific temperature and pH. After 2 h post incubation, the phage suspensions at different temperatures were cooled slowly and then placed in an ice-water bath. The phage suspensions at different pH were neutralized to pH 7. The stability of phage was determined by titration. Phage incubated at pH 7 and 25 °C were used as a control. The experiments were undertaken independently in duplicate with duplicate plaque assay.

### UV radiation stability

The stability of phage under UV radiation was tested according to a previous report^33^. Briefly, phage lysate was diluted to a final concentration of 10^8^ PFU/mL in a final volume of 10 mL of SM buffer. The samples were added into open Petri dishes on ice, placed 30 cm away from the light source, and then exposure to the UV-C light for the indicated time points. The intensity of the UV-C light was 1.722 ± 0.1120 w/cm^2^ (SYLVANIA ultraviolet G30W lamp)^71^. Phage lysate was collected for titration by double agar overlay plaque assay every 10 min for 1 h. The experiments were undertaken independently in duplicate with duplicate plaque assay.

### Long-term stability

The soft-agar overlay method was used to quantitate the long-term stability of the phage^72,73^. The phage was stored at 4 °C, for over one year and the stability was measured monthly. The experiments were undertaken independently in duplicate with duplicate plaque assay.

### Whole genome characterization and analysis

Whole genome sequencing was carried out commercially on the Illumina sequencing platform, (Macrogen Inc., Seoul, South Korea), using de novo assembly analysis. The genome of phage KP1801 was extracted and DNA quality control was performed followed by library construction. The TruSeq Nano DNA library preparation kit was used for genome sequencing, according to the manufacturer’s instructions. The sequence library was prepared by random fragmentation of DNA sample which were then ligated to 5′- and 3′-adapters. Adapter-ligated fragments were amplified using PCR followed by gel purification and sequencing. Sequencing data were converted into raw data for analysis. Filtered reads were used for de novo assembly in a contig and the de novo assembly was performed by various k-mer using SPAdes. The locations of protein coding sequences, tRNA genes, and rRNA genes were predicted by Prokka (v1.12) and the functions were then annotated by BLAST.

All pairwise comparisons of the nucleotide sequences were conducted using the Genome-BLAST Distance Phylogeny (GBDP) method^74^ under settings recommended for prokaryotic viruses^75^. The resulting intergenomic distances were used to infer a balanced minimum evolution tree with branch support via FASTME including SPR post-processing^76^ for each of the formulas D0, D4, and D6, respectively. Branch support was inferred from 100 pseudo-bootstrap replicates each. Trees were rooted at the midpoint and visualized with FigTree. Taxon boundaries at the species, genus, and family level were estimated with the OPTSIL program^77^, using the recommended clustering thresholds^75^ and an F value (fraction of links required for cluster fusion) of 0.5^78^.

### Phylogenetic tree analysis of specific genes

Neighbor-joining phylogenetic trees of phage KP1801 were constructed based on the capsid maturation protease, phage tail fiber proteins, endolysin, and holin. The sequences were aligned using MUSCLE in the MEGA-X software and bootstrap percentage analyses were based on 1,000 replications. Sequence identity matrix was calculated by BioEdit.

### Analysis of phage structural proteins

Total phage proteins were extracted with the addition of modified lysis buffer based on Laemmli buffer (50 mM Tris-HCl (pH 6.8), 2% SDS, 1 mM EDTA, 1 mM DTT) in a ratio 1:1 followed by incubation on ice for 30 min. The samples were boiled at 100 °C for 10 min. Protein concentrations were quantified by the Lowry method. For SDS-PAGE analysis, 5 micrograms of protein were separated by electrophoresis through 12.5% polyacrylamide gels run at 200 V for 1 h followed by silver staining. Spectra Multicolor Broad Range Protein Ladder (2663, Thermo Fisher Scientific, Waltham, MA) was used as a molecular weight standard.

For shotgun proteome analysis, phage protein samples were subjected to in-gel digestion. Five micrograms of protein were re-suspended in a mixture of 40% acrylamide/bisacrylamide, 1.5 Tris-HCl (pH 8.8), 10% SDS and 10% ammonium persulfate followed by addition of tetramethylethylenediamine (TEMED). The mixture was immediately centrifuged at 10,000×*g* for 5 min and subsequently incubated for 5 min at room temperature, leading to gel formation. Sterile milliQ water was added into the gel followed by incubation for 5 min at room temperature. After removing the water, the 100% acetonitrile (ACN) was added to the gel followed by incubation for 5 min. This step was repeated three times and the gels were then dried for 30 min at room temperature. Subsequently, 10 mM dithiothreitol (DTT) in 10 mM NH_4_HCO_3_ was added to reduce disulfide bonds. Samples were then incubated for 1 h at 56 °C followed by removal of the solution. One hundred millimolar iodoacetamide (IAA) in 10 mM NH_4_HCO_3_ was added and samples were incubated in the dark for 1 h at room temperature. IAA/NH_4_HCO_3_ was removed and then samples were incubated in 100% ACN at room temperature for 5 min. These steps were repeated three times. After removing ACN, the gel was allowed to dry for 15 min followed by the addition of 10 mM NH_4_HCO_3._ The gels were ground and then rehydrated by addition of 100% ACN followed by removal of ACN. The gel was allowed to dry for 15 min and then proteins in the gel digested with trypsin (Promega, Madison, WI) in 10 mM NH_4_HCO_3_ at 37 °C overnight. Subsequently 100% ACN was added followed by incubation for 5 min at room temperature. The digested proteins in the supernatant were transferred into new Eppendorf tubes. For the original Eppendorf tubes, 0.1% formic acid was added into the tubes which were then incubated for 5 min at room temperature, after which 100% ACN was added into the tubes which were incubated at room temperature for 5 min. The supernatant was transferred into the new Eppendorf tubes contained digested proteins following which the samples were then dried at 37 °C overnight. The dried samples were resuspended in 0.1% formic acid and then collected in small vials with screw caps for proteome analyses.

### Liquid chromatography-tandem mass spectrometry (LC/MS–MS)

The peptide samples were injected into an HCTUltra LC–MS system (Bruker Daltonics Ltd; Hamburg, Germany) coupled with an UltiMate 3000 LC nano System (Thermo Fisher Scientific, Waltham, MA). An electrospray was flown into a nanocolumn at the flow rate of 300 nL/min. The injections were spiked with 200 fmol tryptic digested peptides of bovine serum albumin, as internal standards. Solution A (0.1% formic acid) and solution B (80% acetonitrile and 0.1% formic acid) were run for 40 min. Subsequently, the peptides in the column were eluted by a linear gradient of 10–70% and 90% solution B, respectively. The final elution of 10% solution B at 20 min was carried out to remove any remaining salt. The injections were undertaken in triplicate. For analysis, proteins were quantitated by DeCyder MS Differential Analysis software (DeCyderMS, GE Healthcare, Chicago IL) followed by conversion of LC–MS raw data. Automated peptide detection, charge state assignments, and quantitation based on the peptide ions signal intensities in MS mode were processed using the PepDetect module. The proteins were identified from MS/MS peptide mass values by Mascot software (Matrix Science, London, UK) and the data was searched against the NCBI database for protein identification.

### Biofilm treatment with phage KP1801

To evaluate the anti-ESBL-KP biofilm potential, the effect of phage on biofilm formation by ESBL-KP was determined by the co-incubation of phage and bacteria at 37 °C without agitation for 24 and 48 h as described previously^66,79–82^. Briefly, ESBL-KP in TSB were added into a flat-bottomed 96-well microtiter plate (Thermo Fisher Scientific Inc., Waltham, MA) at concentrations of 10^8^ CFU/mL in 100 μL. The wells were then supplemented with 100 μL of the indicated number of phage (10^1^–10^8^ PFU/well) followed by incubation at 37 °C for the indicated times. For established biofilm, one hundred microliters of the bacterial culture at concentrations of 10^8^ CFU/mL was seeded into a flat-bottomed 96-well microtiter plate and then supplemented with 100 μL of TSB followed by incubation at 37 °C. At 24 and 48 h post incubation, the medium and planktonic cells were gently removed and the wells were then washed twice with TSB. Phage diluted in TSB (10^1^–10^8^ PFU/well) were added into the wells. After exposure for 24 h at 37 °C, the contents of the wells were discarded and the wells were then washed twice with PBS. At the indicated time-points, biofilms biomass of both biofilm formation and preformed biofilm was quantitated by crystal violet staining. The wells were air-dried and then stained with 200 μL of 0.1% crystal violet for 30 min. The excess crystal violet was removed by washing the wells with PBS four times and wells were air dried for 1 h. Bound crystal violet was then solubilized using 200 μL of 95% ethanol and the optical absorbance was quantified at 600 nm using a microplate absorbance reader.

For determination of biofilm viable cell numbers by colony count, the medium was removed and the wells were then washed with TSB. The biofilm cells were resuspended in 100 µL of PBS (pH 7.4) by vigorous pipetting. The suspended biofilm was serially diluted tenfold in PBS and the number of biofilm viable cells was determined in counting colony forming units on TSA plates after overnight incubation at 37 ℃. The experiments were undertaken independently in duplicate with duplicate assay.

### Scanning electron microscopy (SEM)

Preformed biofilm was used as a model. Biofilms were formed on glass coverslips placed at the bottom of a 12 well plate for 48 h and then treated with phage at 10^5^ and 10^9^ PFU/well. At 24 h post incubation, the cells were washed twice with PBS. Following 2 h fixation with 2.5% glutaraldehyde prepared in 0.1 M PBS (pH 7.4) at room temperature, the fixed cells were dehydrated in a series of ice cold ethanol washes (30% v/v, 40% v/v, 50% /v, 60% v/v, 70% v/v, 80% v/v, 90% v/v, and absolute ethanol). The samples were dehydrated and dried in a critical point dryer with liquid CO_2_ followed by coating with gold particles. The amount of biofilm and cell morphological changes were assessed under a field emission scanning electron microscope (Apreo, Thermo Fisher Scientific) with an accelerating voltage of 5 kV.

### *Galleria mellonella* phage therapy assays

*Galleria* *mellonella* (Greater wax moth or honeycomb moth) larvae were used as a model for evaluating the potential of phages against infection^66^. Healthy larvae with approximately 300 mg weight were selected and the larvae were then surface-decontaminated with a cotton swab containing 70% ethanol. A set of 10 larvae per group was used. Twenty microliters of inoculum containing a series of tenfold serial dilutions from 5 × 10^4^ to 5 × 10^7^ CFU/mL of ESBL-KP diluted in 10 mM PBS pH 6.5 was injected into *G.* *mellonella* larvae through the last left pro-leg followed by incubation at 37 °C in darkness under a humidified atmosphere, with food. Only PBS injection was used as a negative control. Pigmentation and mortality of larvae were recorded daily for 5 days. If larvae moved, responded to physical stimuli with a pipette tip and showed no sign of melanization, it was considered alive. If there was no movement or the larvae was melanized, it was considered as dead. A half-maximum lethal dose (LD50) was calculated for the treatment models. The number of bacteria in larvae was evaluated by counting CFU at various time points.

For the prophylactic treatment model of phage, larvae were injected with phage followed by bacteria infection. Briefly, larvae were inoculated with 20 μL of a phage at MOI of 1, 10, 100 and 1,000 into the haemolymph. At 2 h post-infection, the larvae were then injected with 20 μL of inoculum containing the LD50 dose of ESBL-KP in PBS buffer on the opposite side of the larvae to the phage inoculation. To determine the therapeutic efficacy of phage, larvae were infected with bacteria followed by phage injection. Larvae were infected with 20 μL of ESBL-KP suspension into the larval haemolymph and incubated for 2 h. The larvae were then injected with 20 μL of phage at MOI of 1, 10, 100 and 1,000 on the opposite side to the bacterial injection site.

For both treatment models, larvae were placed into Petri dishes and then incubated at 37 °C. The pigmentation, mobility, and survival rate of the larvae were recorded daily. For negative controls, groups of larvae injected with PBS and phage with an MOI of 1,000 were used. The positive control group of larvae were infected with only ESBL-KP. A total of 3 independent groups (n = 10 larvae per group) were used for each model. The experiment was undertaken independently in duplicate. At day 5 post infection, larvae were ground in ice-cold PBS and the viable bacteria were serially diluted tenfold and then spotted onto TSA plates. The number of bacteria was determined by counting CFU after overnight incubation. The phage recovered from larvae was also quantified. Mixtures were centrifuged at 6,000×*g* for 10 min at 4 °C. The supernatant was filtered through a 0.22 μm pore filter and the phage was quantitated by plaque assay.

### Statistical analyses

All data was analyzed using the GraphPad Prism 8 program (GrapPad Software, San Diego, CA). Statistical analysis of significance was also undertaken using the GraphPad Prism program by unpaired T-test with a *p* < 0.05 for significance.

## Nucleotide sequence accession number

The complete genome sequence was submitted into Genbank and the nucleotide sequence accession number is MN783016.1.

## Supplementary information

Supplementary file1.

Supplementary file2.

Supplementary file3.
