# Supplementary material for: Characterization of extended-spectrum-β-lactamase producing Klebsiellapneumoniae phage KP1801 and evaluation of therapeutic efficacy in vitro and in vivo
Source: Sci Rep. 2020 Jul 16;10:11803. doi: 10.1038/s41598-020-68702-y (PMC7367294; doi:10.1038/s41598-020-68702-y)
Supplement: Supplementary file 1 — Supplementary file1. [file 41598_2020_68702_MOESM1_ESM.docx]

**Supplementary Figure S1.** Analysis of structural proteins of phage KP1801. **Supplementary Figure S2.** Bacterial counts from infected *G. mellonella.* Experiments were undertaken independently in triplicate. The data show the mean±SD.

**Supplementary Table S1** Antimicrobial susceptibility testing of ESBL-KP isolates to ampicillin (AMP), cefotaxime (CAZ), ceftazidime (CTX), ciprofloxacin (CIP), gentamicin (GEN), imipenem (IMP), and meropenem (MER). **Supplementary Table S2** Host range infection of phage KP1801. + able to produce lytic zone, - was unable to produce lytic zone. **Supplementary Table S3** BLASTp results for protein sequence analysis of phage KP1801. **Supplementary Table S4** Proteomic of phage KP1801 by total shotgun proteome analysis.
