# Supplementary material for: Characterization of extended-spectrum-β-lactamase producing Klebsiellapneumoniae phage KP1801 and evaluation of therapeutic efficacy in vitro and in vivo
Source: Sci Rep. 2020 Jul 16;10:11803. doi: 10.1038/s41598-020-68702-y (PMC7367294; doi:10.1038/s41598-020-68702-y)
Supplement: Supplementary file 3 — Supplementary file3. [file 41598_2020_68702_MOESM3_ESM.pdf]

Characterization of extended-spectrum-*β*-lactamase producing *Klebsiella pneumoniae* phage KP1801 and evaluation of therapeutic efficacy *in vitro* and *in vivo*

Phitchayapak Wintachai[1*], Ampapan Naknaen[2], Jirapath Thammaphet[1], Rattanaruji Pomwised[2], Narumon Phaonakrop[3], Sittiruk Roytrakul[3], Duncan R Smith[4]

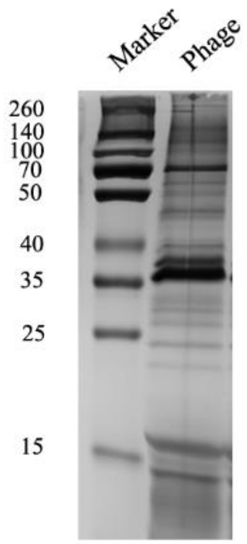

Supplementary Figure S1. Analysis of structural proteins of phage KP1801.

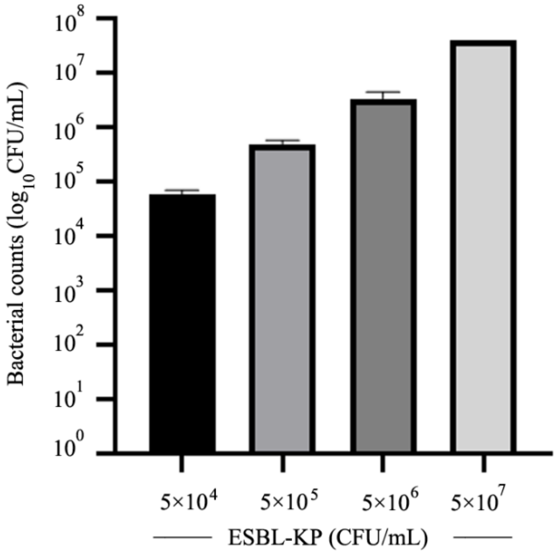

Supplementary Figure S2. Bacterial counts from infected *G.mellonella.* Experiments were undertaken independently in triplicate. The data show the mean±SD.
